# Clinical outcomes of single blastocyst transfer with machine learning guided noninvasive chromosome screening grading system in infertile patients

**DOI:** 10.1186/s12958-024-01231-9

**Published:** 2024-05-23

**Authors:** Xiaoxi Li, Yaxin Yao, Dunmei Zhao, Xiufeng Chang, Yi Li, Huilan Lin, Huijuan Wei, Haiye Wang, Ying Mi, Lei Huang, Sijia Lu, Weimin Yang, Liyi Cai

**Affiliations:** 1 Reproductive Medicine Department of Hebei Maternity Hospital, Shijiazhuang, China; 2 Department of Clinical Research, Yikon Genomics Company, Ltd., Suzhou, China

**Keywords:** Noninvasive chromosome screening-artificial intelligence (NICS-AI), Single blastocyst transfer, Embryo selection, Intracytoplasmic single sperm injection, Infertile

## Abstract

**Background:**

Prospective observational studies have demonstrated that the machine learning (ML) -guided noninvasive chromosome screening (NICS) grading system, which we called the noninvasive chromosome screening-artificial intelligence (NICS-AI) grading system, can be used embryo selection. The current prospective interventional clinical study was conducted to investigate whether this NICS-AI grading system can be used as a powerful tool for embryo selection.

**Methods:**

Patients who visited our centre between October 2018 and December 2021 were recruited. Grade A and B embryos with a high probability of euploidy were transferred in the NICS group. The patients in the control group selected the embryos according to the traditional morphological grading. Finally, 90 patients in the NICS group and 161 patients in the control group were compared statistically for their clinical outcomes.

**Results:**

In the NICS group, the clinical pregnancy rate (70.0% vs. 54.0%, *p* < 0.001), the ongoing pregnancy rate (58.9% vs. 44.7%, *p* = 0.001), and the live birth rate (56.7% vs. 42.9%, *p* = 0.001) were significantly higher than those of the control group. When the female was ≥ 35 years old, the clinical pregnancy rate (67.7% vs. 32.1%, *p* < 0.001), ongoing pregnancy rate (56.5% vs. 25.0%, *p* = 0.001), and live birth rate (54.8% vs. 25.0%, *p* = 0.001) in the NICS group were significantly higher than those of the control group. Regardless of whether the patients had a previous record of early spontaneous abortion or not, the live birth rate of the NICS group was higher than that of the control group (61.0% vs. 46.9%; 57.9% vs. 34.8%; 33.3% vs. 0%) but the differences were not statistically significant.

**Conclusions:**

NICS-AI was able to improve embryo utilisation rate, and the live birth rate, especially for those ≥ 35 years old, with transfer of Grade A embryos being preferred, followed by Grade B embryos. NICS-AI can be used as an effective tool for embryo selection in the future.

**Supplementary Information:**

The online version contains supplementary material available at 10.1186/s12958-024-01231-9.

## Background

Preimplantation genetic testing for aneuploidy (PGT-A) is a clinically used genetic screening test for chromosomal ploidy of embryos, which can select normal blastocysts for transfer, thereby decreasing the miscarriage rate of patients, increasing the live birth rate, and shortening the time to achieve pregnancy [1, 2]. However, PGT-A relies on invasive trophectoderm biopsy, which may affect embryo implantation, development, and even pose a risk to the long-term safety of the offspring [3–5]. In addition, PGT-A reports copy number variations (CNVs) in terms of euploidy and aneuploidy, with euploid embryos being selected for transfer, mosaic and aneuploid embryos being not selected for transfer. This reporting method may make some patients who are advanced in age or who obtain a low number of blastocysts have no euploid embryos to be transferred in the present cycle, resulting in a waste of embryos. In recent years, some studies have begun to focus on the clinical outcomes of mosaic embryos, and it has been demonstrated that a low percentage of mosaic embryos can achieve a good rate of live births after transfer [6, 7].

In 2013, Stigliani et al. reported it was discovered that embryos release cell-free DNA (cfDNA) into the culture medium during in vitro culture, and that the use of cfDNA for the detection of embryonic chromosomal ploidy became possible [8]. In 2016, Xu et al. developed a noninvasive chromosome screening (NICS) technique based on cfDNA, 42 clinical samples were subjected to this NICS testing, complete genome-wide chromosomal ploidy information was obtained, and after transferring normal embryos, live births were successfully obtained in 5 out of 7 patients [9]. In contrast to the invasive trophectoderm (TE) biopsy, sample collection for NICS is noninvasive and easy to obtain. Therefore, several teams have then studied the sampling method, accuracy, and clinical application of NICS. Several studies have thus far demonstrated that NICS results are consistent with whole embryos up to 82.0-100% [10–12], which is comparable to the performance of TE biopsy-based PGT-A assay [13]. A single-center clinical study conducted by our research team in 2019 demonstrated that NICS could improve the clinical pregnancy rate and reduce the miscarriage rate in patients with recurrent miscarriages, recurrent implantation failures, and abnormalities of chromosome copy number [14]. In 2019, Huang et al. suggested in their study that 60% should be used as the threshold for mosaicism in NICS, which resulted in a detection accuracy of NICS that was even higher than that of TE-biopsy PGT-A [10]. Although, threshold-based CNV reporting of NICS can reduce the false-positive rate, and improve the clinical pregnancy, live-birth rates in the patients who transferred embryos, there is still a possibility of embryo wastage, especially in the case of the low percentage of mosaic.

In order to solve the problem of embryo wastage, Chen et al. established a noninvasive chromosome screening-artificial intelligence (NICS-AI) model using machine learning algorithms, which classifies blastocysts into three grading levels of A, B, and C according to the embryo aneuploidy results, and demonstrated that patients transferred with grade A/B embryos can obtain a higher live birth rate via an observational clinical trial, which validated the embryo selection using the NICS-AI grading system [15]. The performance of the NICS-AI grading system for embryo selection was verified, and the embryo utilisation rate was also increased from 57.9 to 78.8%. However, this study was observational and didn’t select the patients’ embryos according to NICS-AI grading results.

In this study, we recruited patients who visited our centre between October 2018 and December 2021, and underwent single blastocyst transfer for the first time. Grade A and B embryos using the NICS-AI grading method for embryo selection were selected to be transferred to these patients, and these patients were classified as the NICS group. The patients transferred with embryos that were selected according to traditional morphology grading methods, were classified as a control group. By comparing the clinical outcomes of these two groups of patients, we investigated whether NICS-AI could be used as a powerful tool for embryo selection. In addition, we further exploited the potential benefit cohort of the NICS-AI grading method by further analyzing the patients’ age and the number of previous miscarriages.

## Methods

### Study subjects

This study is a single-centre prospective interventional clinical study. Patients who visited Hebei Maternity Hospital between October 2018 and December 2021 were recruited. Follow up regarding the primary outcome was completed in April 2023.

Inclusion criteria: the female patient was 22–40 years old, underwent intracytoplasmic sperm injection (ICSI) with ≥ 2 blastocysts, agreed to the first transfer as a single blastocyst transfer, and the transferred embryos were retrieved from a single cryopreservation.

Exclusion criteria: patients with chromosomal abnormalities, uterine anomalies, and endometrial thickness < 7 mm. Patients were recruited in the NICS group and the control group in the ratio of 1:2 numbers. This study was carefully reviewed and approved by the Medical Ethics Committee of Hebei Maternity Hospital (Number: 20180001). All patients provided informed consent to participate in this study. The number of Chinese Clinical Trial Registry was ChiCTR2300072566.

### Oocytes retrieval and granulosa cell removal

According to the patient’s case, ovulation was stimulated by standard antagonist and progestin primed ovarian stimulation (PPOS), and the dose of gonadotropin was adjusted according to the patient’s ovarian response, hormone level and follicle size, and when the follicle diameter and hormone level reached the standard of triggering, the dose of 5,000 to 8,000 IU human chorionic gonadotrophin (HCG) or 0.1 mg gonadotropin-releasing hormone agonist (GnRHa) combined with 4,000 IU HCG was given, and the triggering was performed egg retrieval was performed 37 h later under the guidance of vaginal ultrasound. One to two hours after egg retrieval, oocytes were treated with hyaluronidase and blown and washed three times to remove granulosa cells.

### Embryo culture and sample collection

After ICSI, fertilisation was checked at 16–18 h and two pronuclei and two polar bodies were clearly observed. On the afternoon of the second day after confirmation of fertilization, the granulosa cells of the embryos were again removed, blastocyst culture medium was replaced and the embryos continued to be cultured in the new drops. On the afternoon of the fourth day after fertilization, the blastocyst culture medium was replaced again and the embryos were washed three times, the volume of the culture medium was about 25 µl. These operations were effective in removing the contamination of maternal DNA. When the blastocysts developed to the standard of freezing, the blastocysts were cryopreserved as single blastocysts using the vitrification freezing method, and 20 µl culture medium of the corresponding blastocysts was collected into the RNase/DNAase-free PCR tubes, which containing 5 µl of preservation solution.

### Morphologic grading of blastocysts

Before the blastocysts were frozen, the blastocysts were graded according to the Gardner and Schoolcraft morphological grading system [16, 17]. , which assessed the three components of blastocyst expansion, inner cell mass and trophectoderm. For the control group, the embryos were selected based on morphological grading and were transferred with single blastocysts. According to current expert consensus in China [18], D5 or D6 blastocysts graded as AA, AB, BA, or BB are considered High-quality blastocysts. Those graded as AC, BC, CA, or CB, as well as high-quality blastocysts, can be defined as Usable blastocysts. High-quality blastocysts such as AA, AB, BA, and BB were prioritized for transfer, followed by AC, BC, CA, and CB blastocysts.

### Whole genome amplification, library prep and sequencing

Whole genome amplification (WGA) and next-generation sequencing (NGS) library preparation was performed on the collected blastocyst cultures using the NICSInst™ (Xukang Medical Technology (suzhou) Co., Ltd) library kit according to the instructions [11, 13]. A 10ul spent culture medium was pipetted from the sample preservation tube for WGA, and then the library was built for the amplified products. Quality control of NGS libraries was performed using Qubit 3.0 (Qubit® dsDNA HS Assay Kit, Thermo Fisher Scientific) and 1.5% agarose gel electrophoresis. After mixing the samples according to the aliquots, sequencing was performed using the Illumina platform, and approximately 2 M sequencing reads were obtained for each library.

### Copy number variation (CNV) analysis and a.i. grading system

Data were analysed and visualised using ChromGo™ analysis software (Xukang Medical Technology (suzhou) Co., Ltd) [19]. High-quality reads were counted along the entire genome with a bin size of 1 Mb, and reads were normalized by GC and reference datasets, and the binary segmentation algorithm (CBS) was used for the detection of CNV fragments.

As described in the previous paper [15], the NICS-AI model has been established. The NICS-AI model was an artificial intelligence algorithm using R package caret 6.0–86. The model utilized the Random Forest (RF) machine learning algorithm, with whole embryo CNV results as the gold standard, to develop a copy number pattern in the blastocyst culture media associated with chromosomal euploidy or aneuploidy.

The machine learning methods trained on the following 11 features: 10 M-resolution CNV result, 10 M-resolution CNV result redefined by 50% mosaicism threshold, arm-resolution CNV result, arm-resolution CNV result redefined by 50% mosaicism threshold, whole chromosome-resolution CNV result, whole chromosome-resolution CNV result redefined by 50% mosaicism threshold, euploidy number with different resolution result, abnormal chromosome number, highest abnormal mosaicism proportion, largest abnormal fragment size corresponding to the highest mosaicism proportion, and presence of sex chromosome abnormality or not [15].

The NICS-AI model analysed these features from the sequencing results of the culture media to predict embryo euploidy probabilities, categorising them into ≥ 0.94, 0.7–0.94, and ≤ 0.7 as grades A, B, and C. The recommended embryos to be transferred were A and B-grade. The patients in the NICS group were transferred with A-grade blastocysts in preference to B-grade.

### Embryo thawing and transfer

The embryos were thawed using the commercial thawing kit (Kitazato, Japan), and after thawing, the embryos were transferred according to the clinical routine.

### Follow-up on clinical outcomes

The main clinical outcome was live birth rate of the first single blastocyst transfer. At 14 days after blastocyst transfer, the patient’s hCG value was measured. Clinical pregnancy was the presence of at least one gestational sac in the uterine cavity as determined by ultrasound at 28–30 days after transfer. An ongoing pregnancy was defined as a detectable fetal heart at week 12 of gestation. Live birth was defined as delivery of 1 live infant with a gestational age greater than 28 weeks. The following information was collected within 2 weeks of delivery: birth sex, birth weight, and neonatal score.

### Statistical analysis

Data were analysed by normal distribution. Values that matched normal distribution were shown as mean and standard deviation, and t-tests were used for comparison between groups, while non-normally distributed data were shown as median (Q1-Q3), and Mann-Whitney tests were used between groups. The chi-square test was used to assess the comparison between groups of the component ratios or rates (%). Multiple logistic regression analysis was used to test clinical pregnancy rates, ongoing pregnancy rates, and live birth rates between groups. The demographic data, including female age, male age, number of previous early spontaneous abortions, indication, types of infertility, gonadotropins (Gn) dosage, and Gn days were incorporated into the model and used to analyze the odds ratio (OR) for clinical outcomes.

Statistical analysis was performed using SPSS, V.25.0 (SPSS, Chicago, Illinois, USA), and statistical differences were considered statistically significant at *p* < 0.05.

## Results

### Study workflow

According to inclusion/exclusion criteria and the 1:2 ratio of the NICS group and the control group, 95 patients were in the NICS group and 190 patients were in the control group. In the NICS group, 1 patient had all embryos tested at grade C; 2 patients abandoned the transfer for their own medical reasons; and 2 patients underwent double blastocyst transfer. In the control group, 10 patients chose to undergo a fresh cycle of embryo transfer; 8 patients forfeited the transfer for their own medical reasons; 3 patients underwent double blastocyst transfer; and 8 patients withdrew informed consent.

**Fig. 1:**
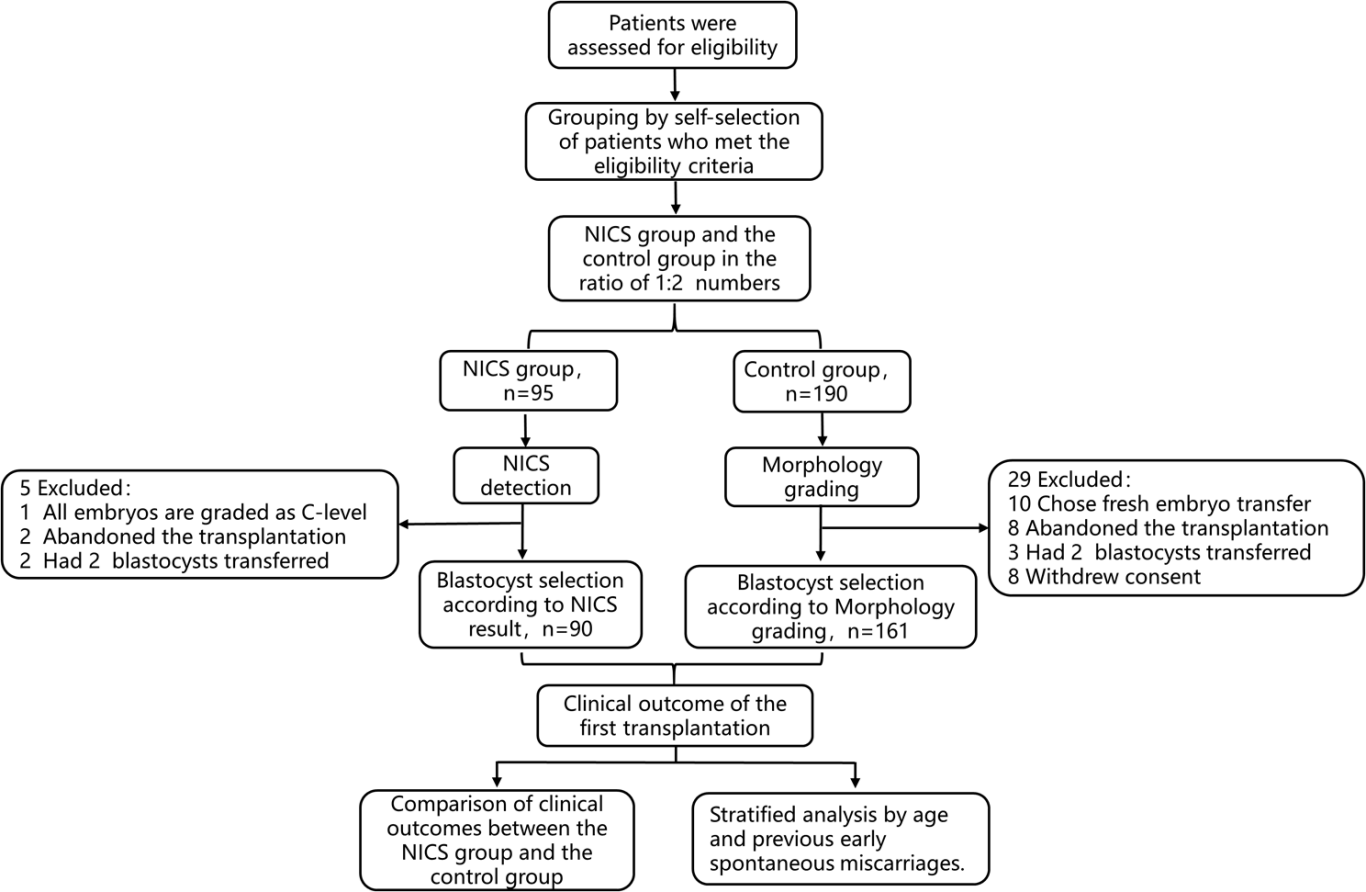
Flowchart of the study. The NICS group and the control group included 90 and 161 patients for analysis, respectively

The final NICS group included in the analysis had 90 patients who were transferred embryos according to the NICS-AI grading method. In the control group there were 161 patients who were transferred with single blastocyst embryos according to morphology grading method. These 251 patients were followed up for clinical outcome of the first transfer. The study’s workflow is shown in Fig. 1.

### CNV analysis of NICS group

**Fig. 2:**
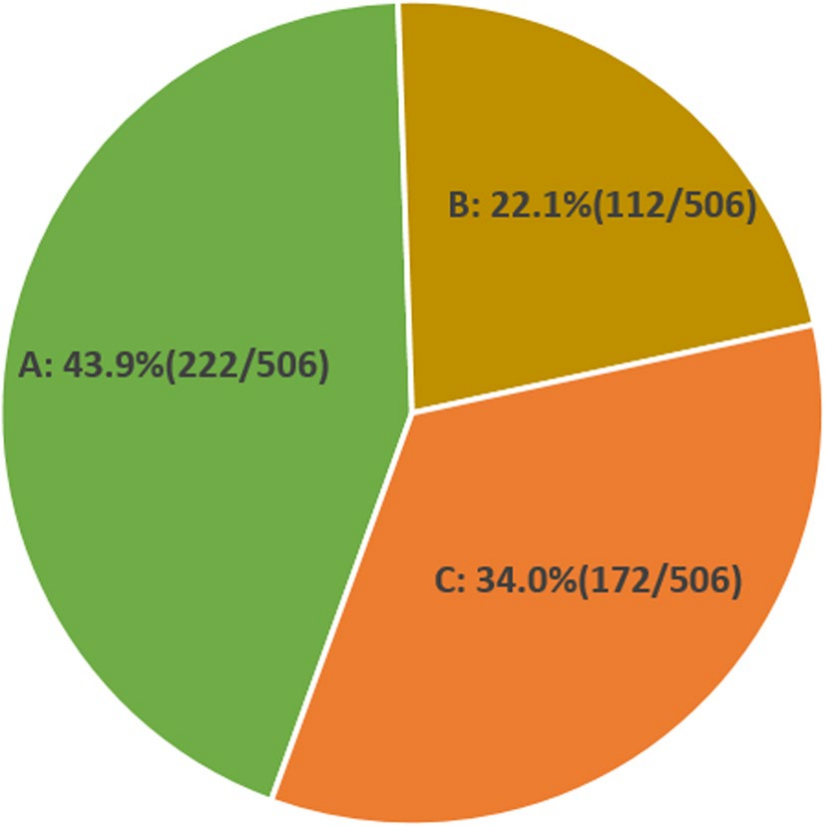
Proportion of embryos with different NICS-AI grades in the NICS group

To investigate the utilisation of embryos in the NICS group of patients, 506 blastocysts from 90 patients in the NICS group were analysed (Fig. 2). The results showed that the number of embryos with A, B, and C grades were 222, 112, and 172, respectively, and 66.0% of the total number of embryos had A + B grades (334/506).

### Baseline characteristics and clinical outcomes of the patients

The baseline characteristics of the patients and the clinical data of the oocyte retrieval cycles are presented in Table 1. The female age was significantly higher in the NICS group than in the control group (35.1 ± 4.2 vs. 32.5 ± 4.9, *p* < 0.001), and the percentage of patients with a history of spontaneous abortion was significantly higher in the NICS group than in the control group (34.4% vs. 19.3%, *p* = 0.007). There was also a significant difference between the NICS group and the control group in terms of male age, indication, and types of infertility (*p* < 0.05), and for the rest of the baseline characteristics, the differences between the two groups were not statistically significant. For the data in the oocyte retrieval cycles, the Gn dosage and Gn days exhibited statistical differences in the NICS and control groups (*p* < 0.05). The rest of the data was not significantly different in both groups.

**Table 1:** Baseline characteristics of patients and clinical data from the oocyte retrieval cycle

	NICS group	Control group	*P* value
**Characteristic**			
No. of patients	90	161	
Female Age (y)	35.1 ± 4.2	32.5 ± 4.9	<0.001
Female BMI (kg/m^2^)	24.3 ± 3.5	24.5 ± 4.3	0.678
Male Age (y)	34.6 ± 4.2	33.1 ± 4.9	0.021
Male BMI (kg/m^2^)	26.5 ± 3.5	26.1 ± 4.2	0.412
Infertility duration (y)	2.0(1.0–6.0)	3.0(2.0–5.0)	0.452
**No. of previous failed transfer cycles**			
0	76.7%(69/90)	72.7%(117/161)	0.663
1–2	20.0%(18/90)	21.7%(35/161)	
≥3	3.3%(3/90)	5.6%(9/161)	
**No. of previous early spontaneous miscarriages**			
0	65.6%(59/90)	80.7%(130/161)	0.015
1	21.1%(19/90)	14.3%(23/161)	
≥2	13.3%(12/90)	5.0%(8/161)	
**Indication**			
Male factor	13.3%(12/90)	37.9%(61/161)	<0.001
Female factor	76.7%(69/90)	44.1%(71/161)	
Male and female factor	10.0%(9/90)	18.0%(29/161)	
**Types of infertility**			
Primary infertility	22.2%(20/90)	49.1%(79/161)	<0.001
Secondary infertility	77.8%(70/90)	50.9%(82/161)	
**Oocyte retrieval cycle**			
AMH (ng/mL)	2.9(1.7–4.9)	3.1(1.7–5.1)	0.652
**Laboratory testing**			
FSH (IU/L)	7.5 ± 2.1	7.9 ± 2.6	0.243
LH (IU/L)	4.8 ± 2.6	4.6 ± 2.6	0.562
E2 (pg/ml)	44.0(34.1–57.0)	39.8(28.0-53.6)	0.066
P (nmol/L)	0.6(0.4–0.9)	0.59(0.4–0.8)	0.838
AFC	15.4 ± 6.9	14.3 ± 6.5	0.199
**Ovarian stimulation protocol**			
GnRH-ant protocol	74.4%(67/90)	70.8%(114/161)	0.128
PPOS protocol	23.3%(21/90)	20.5%(33/161)	
Others protocol	2.2%(2/90)	8.7%(14/161)	
Gn dosage	2077.5 ± 674.9	1856.0 ± 617.8	0.009
Gn days	10.0 ± 1.8	9.5 ± 1.8	0.023
No. of retrieval oocytes	13.2 ± 6.0	12.2 ± 6.9	0.266
No. of MII oocytes	11.4 ± 6.1	10.5 ± 6.1	0.307
No. of successful oocyte fertilisations	11.0 ± 6.0	10.3 ± 6.0	0.388

BMI: Body mass index; AMH: anti-Müllerian hormone; FSH: Follicle-stimulating hormone; LH: Luteinizing hormone; E2: Estradiol; P: Progesterone; AFC: Antral follicle count; PPOS: Progestin primed ovarian stimulation; GnRH: Gonadotrophin-releasing hormone; Gn: gonadotrophins; MII, Mature oocyte

Clinical outcomes of first transfer in the NICS and control groups were analysed, and as shown in Supplementary Table 1, the clinical pregnancy rate (70.0% vs. 54.0%, *p* = 0.013), ongoing pregnancy rate (58.9% vs. 44.7%, *p* = 0.031), and live-birth rate (56.7% vs. 42.9%, *p* = 0.036) in the NICS group were significantly higher than those of the control group. Birth weight and neonatal score were lower in the NICS group than in the control group, but the difference was not statistically significant (weight (g): 3300.5 ± 642.1 vs. 3416.7 ± 481.8, *p* = 0.258; neonatal score: 9.4 ± 1.5 vs. 9.7 ± 1.0, *p* = 0.151). There were no significant differences in the rates of early miscarriage (15.9% vs. 17.2%, *p* = 0.824), mid-to-late-term miscarriage rate (3.2% vs. 3.4%, *p* = 0.927) and preterm birth rate (11.8% vs. 2.9%, *p* = 0.070) between the two groups.

Since the patients in the NICS group and the control group, were statistically different (*p* < 0.05) in terms of female age, male age, number of previous early spontaneous abortions, indication, types of infertility, Gn dosage and Gn days, these variables were taken as independent variables and included in the binary logistic regression analyses. The results showed that there were still significant differences in the clinical pregnancy rate, ongoing pregnancy rate, and live birth rate in the NICS group (adjusted *p* value < 0.05) (Table 2).

**Table 2:** Logistic regression analysis compared the clinical outcomes of the NICS group and control group

Clinical outcomes	NICS group	Control group	Adjusted *P* value ^a^	Adjusted OR (95% CI) ^a^
No. of patients	90	161		
Biochemical pregnancy rate	83.3%(75/90)	73.3%(118/161)	0.010	2.595(1.261 ~ 5.342)
Clinical pregnancy rate	70.0%(63/90)	54.0%(87/161)	< 0.001	3.355(1.739 ~ 6.475)
Ongoing pregnancy rate	58.9%(53/90)	44.7%(72/161)	0.001	3.089(1.631 ~ 5.854)
Live birth rate	56.7%(51/90)	42.9%(69/161)	0.001	2.822(1.513 ~ 5.266)

^a^ Logistic regression analysis adjusted for the female age, male age, number of previous early spontaneous abortions, indication, types of infertility, Gn dosage and Gn days

Furthermore, a head to head pregnancy comparison of blastocysts of NICS and control groups was conducted. Following the domestic consensus on embryo quality definitions [18], the control group’s embryos were classified as High-quality (AA, AB, BA, BB) and Usable (excluding high-quality blastocysts). The results showed that the live birth rate of A-grade embryos in the NICS group was higher than that of High-quality embryos in the control group, but the difference was not statistically significant (63.5% vs. 52.9%, *p* = 0.172). The live birth rate of B-grade embryos in the NICS group was significantly higher than that of Usable embryos (excluding high-quality embryos) in the control group (40.7% vs. 14.3%, *p* = 0.013) (Supplementary Table 2).

### Stratified analysis exploring the factors influencing clinical outcomes

Due to female age, the history of previous early spontaneous miscarriages, there is an impact on the clinical outcome of the patients, and in this study there was a significant difference between the NICS group and the control group. Consequently, we stratified the female age and the history of previous early spontaneous miscarriages.

The results showed that when the female partner was < 35 years old, there was no significant difference between the NICS group and the control group, in terms of biochemical pregnancy rate, clinical pregnancy rate, ongoing pregnancy rate, and live birth rate (*p* = 0.323, *p* = 0.351, *p* = 0.390, *p* = 0.432). When the age of the female partner was ≥ 35 years old, the NICS group had a biochemical pregnancy rate (82.3% vs. 66.1%, *p* = 0.044), clinical pregnancy rate (67.7% vs. 32.1%, *p* < 0.001), ongoing pregnancy rate (56.5% vs. 25.0%, *p* = 0.001) and live birth rate (54.8% vs. 25.0%, *p* = 0.001) that was significantly higher than the control group (Table 3).

**Table 3:** Female age-stratifed comparison of clinical outcomes

Female age	Group	Number of patients	Biochemical pregnancy rate	Clinical pregnancy rate	Ongoing pregnancy rate	Live birth rate
< 35 years old	NICS group	28	85.7%(24/28)	75.0%(21/28)	64.3%(18/28)	60.7%(17/28)
	Control group	105	77.1%(81/105)	65.7%(69/105)	55.2%(58/105)	52.4%(55/105)
	*P* value		0.323	0.351	0.390	0.432
≥ 35 years old	NICS group	62	82.3%(51/62)	67.7%(42/62)	56.5%(35/62)	54.8%(34/62)
	Control group	56	66.1%(37/56)	32.1%(18/56)	25.0%(14/56)	25.0%(14/56)
	*P* value		0.044	<0.001	0.001	0.001

When the results were stratified by history of previous miscarriages, the results showed that the live birth rate was higher in the NICS group than in the control group regardless of whether the patients had a history of early spontaneous miscarriage or not (61.0% vs. 46.9%; 57.9% vs. 34.8%; and 33.3% vs. 0%), but the differences were not statistically significant (Table 4). As the number of miscarriages of the patients increased, the live birth rate improved a little bit more with NICS. When the patients had no history of miscarriage, the live birth rate in the NICS group was 61.0%, which was 14.1% higher than that of the control group. When patients had ≥ 2 previous spontaneous miscarriages, the live birth rate was 33.3% higher in the NICS group than that in the control group (Table 4).

**Table 4:** The number of previous early spontaneous miscarriages-stratified comparison of clinical outcomes

Number of previous early spontaneous miscarriages	Group	Number of patients	Biochemical pregnancy rate	Clinical pregnancy rate	Ongoing pregnancy rate	Live birth rate
0	NICS group	59	88.1%(52/59)	74.6%(44/59)	62.7%(37/59)	61.0%(36/59)
	Control group	130	74.6%(97/130)	57.7%(75/130)	49.2%(64/130)	46.9%(61/130)
	*P* value		0.035	0.026	0.085	0.072
1	NICS group	19	84.2%(16/19)	73.7%(14/19)	63.2%(12/19)	57.9%(11/19)
	Control group	23	69.6%(16/23)	47.8%(11/23)	34.8%(8/23)	34.8%(8/23)
	*P* value		0.305	0.089	0.067	0.134
≥ 2	NICS group	12	58.3%(7/12)	41.7%(5/12)	33.3%(4/12)	33.3%(4/12)
	Control group	8	62.5%(5/8)	12.5%(1/8)	0.0%(0/8)	0.0%(0/8)
	*P* value		1.000	0.325	0.117	0.117

## Discussion

In this study, we compared the clinical outcomes of patients who underwent their first single blastocyst transfer according to the NICS-AI grading method with those who underwent single blastocyst transfers according to traditional morphology grading of preferred embryos. This is the first prospective interventional clinical trial of preferential embryo transfer according to NICS-AI grading. The results of this study showed that the clinical pregnancy rate (70.0% vs. 54.0%, *p* < 0.001), ongoing pregnancy rate (58.9% vs. 44.7%, *p* = 0.001), and live birth rate (56.7% vs. 42.9%, *p* = 0.001) in the NICS group were significantly higher than those in the control group (Table 2), which suggests that the NICS-AI was able to improve the live birth rate of patients through screening the embryos for CNV, which improves the live birth rate of patients. This is consistent with the results of an observational clinical trial conducted by Chen [15]. Xi et al. also reported in their paper that NICS could significantly improve the clinical pregnancy rate in patients with RIF and the live birth rate in patients with RPL compared to the morphology grading method [20].

The previous NICS-AI model was based on the prediction of the embryo euploid probability by Chen et al. The CNV results of embryo culture medium were analysed by machine learning algorithms, and then the embryos were transferred according to the grade order, and the A-grade embryos were preferentially selected to transfer, followed by the B-grade embryos [15]. Through observational clinical trials, we proved that by using the NICS-AI model to select embryos preferentially, the utilisation rate of embryos was able to increase from 57.9% (161/278) to 78.8% (219/278), and the live birth rate of patients who were transferred grade A or B embryos was higher than that of patients who were transferred grade C (50.4% versus 45.3% versus 27.1%). In this study, we strictly evaluated the embryos of patients in the NICS group according to the NICS-AI grading, and out of 506 embryos, 334 embryos, or 66.0% (334/506), with A + B grading (Table 2), showed a high embryo utilisation rate. With the increasing resolution and accuracy of aneuploidy detection technology, the mosaicism of embryos has been demonstrated. It has been found that mosaic embryo transfer can also result in live births [21, 22], and less than 50% of mosaic embryos even have a similar live birth rate to that of euploid embryos (42.6% vs. 43.4%) [6]. While the traditional transfer strategy, i.e., transferring only the euploid embryos, may result in the wastage of a low percentage of mosaic or small fragment abnormal embryos, leading to an increase in the number of cycles with no embryos available for transfer, the NICS-AI model, which selects embryos optimally with embryo grading, greatly improves the utilisation of embryos in the preimplantation genetics screening and improves the transfer rate of the patients. In addition, the standard sampling method in this study minimized the maternal contamination of granulosa cells in the culture medium and reduced false negatives [19].

In addition, considering the significant differences in baseline characteristic age and number of miscarriages between the NICS group and the control group, we performed stratified analyses for female age and number of miscarriages. The results showed that when patients were ≥ 35 years old, the live birth rate was significantly better in the NICS group than in the control group (54.8% vs. 25.0%, *p* = 0.001). However, in patients < 35 years of age, the live birth rate in the NICS group was not significantly higher than the control group (60.7% vs. 52.4%, *p* = 0.432) (Table 4). This suggests that NICS may benefit patients ≥ 35 years old. Age is an important factor influencing the clinical outcome of patients; the probability of chromosomal aneuploidy in women aged 26 ~ 34 years ranges from approximately 20 ~ 31%, and the prevalence of oocyte and embryo aneuploidy increases progressively (34 ~ 75%) when age is ≥ 35 years old [23]. Dang et al. demonstrated that the rate of chromosomal abnormality of embryos was significantly higher in patients with advanced age (≥ 38 years old) than those with age of < 38 years in IVF cycle (54.17% vs. 38.05%, *p* < 0.001) [24]. This contributes to the high rate of miscarriage and low rate of live births in patients of advanced age.

Fang et al. performed noninvasive preimplantation genetic testing for aneuploidy with blastocyst culture media that were from 50 cycles among 45 couples with recurrent implantation failures (≥ 3) or recurrent miscarriages (≥ 3), and the patients achieved a 50% (29/50) clinical pregnancy rate and 27 normal live births [14]. Xi et al. found that in patients with a history of recurrent miscarriages (≥ 2), compared to controls, NICS could significantly improve the rate of sustained pregnancy and live births in patients [20]. In our study, for patients with a history of recurrent miscarriages, although the live birth rate was higher in the NICS group than in the control group (33.3% vs. 0%), the difference was not significant (*p* > 0.05). This may be due to the fact that the study included fewer patients with recurrent miscarriage histories, with only 12 in the NICS group and 8 in the control group. Also for patients with one spontaneous miscarriage, the live birth rate was higher than the control group (57.9% vs. 34.8%), but the difference was not statistically significant, again, probably related to the small sample size. This needs to be further explored in subsequent clinical trials with a larger sample size.Overall, this study demonstrated that NICS-AI grading system was able to improve embryo utilisation in patients, with a preference for transferring A-grade embryos, followed by B-grade embryos. The grading model was also able to improve the first live birth rate in patients undergoing single blastocyst transfer, especially in female ≥ 35 years old. In the future, NICS-AI may be able to serve as a powerful tool for embryo selection and improve clinical outcomes for patients.

### Electronic supplementary material

Below is the link to the electronic supplementary material.

Supplementary Material 1

## Data Availability

The datasets used and/or analyzed during the current study are available from the corresponding author on reasonable request.

## Abbreviations

NICS: Noninvasive Chromosomal Screening
NICS-AI: Noninvasive Chromosome Screening- Artificial Intelligence
PGT-A: Preimplantation Genetic Testing for Aneuploidy
CNV: Copy Number Variation
cfDNA: cell-free DNA
TE: Trophectoderm
ICSI: Intracytoplasmic Sperm Injection
PPOS: Progestin Primed Ovarian Stimulation
HCG: Human Chorionic Gonadotrophin
WGA: Whole Genome Amplification
NGS: Next-Generation Sequencing
Gn: Gonadotrophin
