# Supplementary material for: Clinical outcomes of single blastocyst transfer with machine learning guided noninvasive chromosome screening grading system in infertile patients
Source: Reprod Biol Endocrinol. 2024 May 23;22:61. doi: 10.1186/s12958-024-01231-9 (PMC11112939; doi:10.1186/s12958-024-01231-9)
Supplement: Supplementary file 1 — Supplementary Material 1 [file 12958_2024_1231_MOESM1_ESM.docx]

**Supplementary Table 1.** Comparison of clinical outcomes between the NICS and control group.

| **Clinical outcomes** | **NICS group** | **Control group** | ***P* value** |
| --- | --- | --- | --- |
| No. of patients | 90 | 161 |  |
| Endometrial thickness on the day of embryo transfer (cm) | 9.5 ± 1.5 | 9.9 ± 1.8 | 0.097 |
| Morphological quality of the transferred embryos |  |  | 0.378 |
| High-quality(AA,AB,BA,BB) | 78.9%(71/90) | 73.9%(119/161) |  |
| Usable(Excluding high-quality blastocysts) | 21.1%(19/90) | 26.1%(42/161) |  |
| Biochemical pregnancy rate | 83.3%(75/90) | 73.3%(118/161) | 0.070 |
| Clinical pregnancy rate | 70.0%(63/90) | 54.0%(87/161） | 0.013 |
| Ongoing pregnancy rate | 58.9%(53/90) | 44.7%(72/161） | 0.031 |
| Live birth rate | 56.7%(51/90) | 42.9%(69/161） | 0.036 |
| Early miscarriage rate | 15.9%(10/63) | 17.2%(15/87) | 0.824 |
| Mid-to-late-term miscarriage rate | 3.2%(2/63) | 3.4%(3/87) | 0.927 |
| Preterm birth rate | 11.8%(6/51) | 2.9%(2/69) | 0.070 |
| No. of live births * | 52 | 69 |  |
| Birth weight (g) | 3300.5 ± 642.1 | 3416.7 ± 481.8 | 0.258 |
| Neonatal score | 9.4 ± 1.5 | 9.7 ± 1.0 | 0.151 |

* There was one case of twin births in the NICS group.

**Supplementary Table 2.** The head to head Pregnancy comparison of NICS and control groups.

| **Group** | **Number of patients** | **Biochemical pregnancy rate** | **Clinical pregnancy rate** | **Ongoing pregnancy rate** | **Live birth rate** |
| --- | --- | --- | --- | --- | --- |
| NICS group: A-grade | 63 | 87.3%(55/63) | 76.2%(48/63) | 66.7%(42/63) | 63.5%(40/63) |
| Control group: High-quality (AA,AB,BA,BB) | 119 | 81.5%(97/119) | 64.7%(77/19) | 54.6%(65/119) | 52.9%(63/119) |
| *P* value |  | 0.317 | 0.112 | 0.116 | 0.172 |
| NICS group: B-grade | 27 | 74.1%(20/27) | 55.6%(15/27) | 40.7%(11/27) | 40.7%(11/27) |
| Control group: Usable(Excluding high-quality blastocysts) | 42 | 50.0%(21/42) | 23.8%(10/42) | 16.7%(7/42) | 14.3%(6/42) |
| *P* value |  | 0.047 | 0.007 | 0.026 | 0.013 |
